# Palmar dermatofibroma in a patient with multiple porokeratosis^☆^

**DOI:** 10.1016/j.abd.2021.07.008

**Published:** 2022-09-13

**Authors:** Toshiyuki Yamamoto

**Affiliations:** Department of Dermatology, Fukushima Medical University, Fukushima, Japan

**Keywords:** Dermatofibroma, Palm, Porokeratosis

## Abstract

Although dermatofibromas are not uncommon benign dermal nodules, palms are rarely involved. Herein, a rare case of palmar dermatofibroma was described, which occurred in a patient with porokeratosis.

Dermatofibromas (DFs) are common, benign dermal nodules that frequently occur on the extremities, shoulders, and buttocks of middle-aged women. DFs rarely occur on the digits^1^; palmar involvement is extremely rare.

A 69-year-old male visited our clinic, complaining of multiple brownish plaques on the trunk, heel, and buttocks (Fig. 1). He had Diabetes *Mellitus*, angina, and hypertension. Biopsy taken from the edge of the plaque revealed mild hyperkeratosis and a narrow stack of parakeratotic corneocytes (cornoid lamella), corresponding with the features of porokeratosis. He had been under treatment with topical corticosteroid ointment and etretinate (Tigason^R^) (10‒20 mg/day) for 5 years, but without any significant sufficient improvement. During the course, he complained of a slightly tender nodule on his palm which had appeared 2 months previously. Physical examination revealed a 5-mm sized, brownish firm nodule on the right palm (Fig. 2). Histopathological examination revealed fibrous proliferation in the dermis with the mild acanthosis of the overlying epidermis (Fig. 3). Storiform patterns were not found, and the proliferated fibroblasts were positive for factor XIIIa and CD68, but negative for CD34.

**Figure 1:**
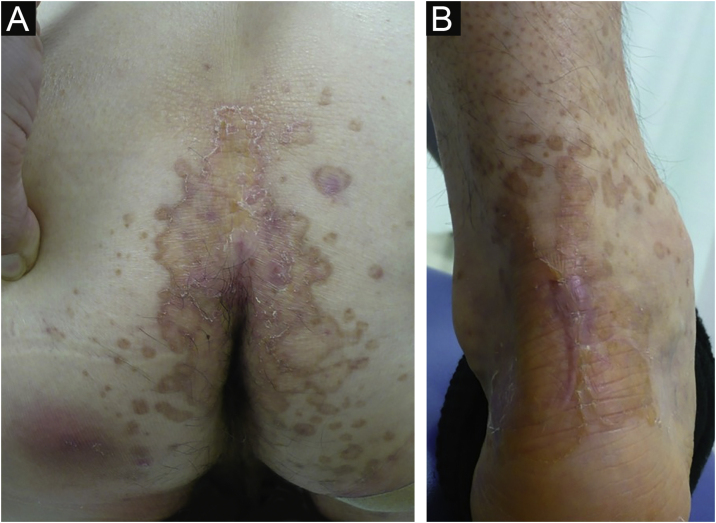
Well-defined brownish plaques of porokeratosis on the buttock (a) and Achilles tendon (b).

**Figure 2:**
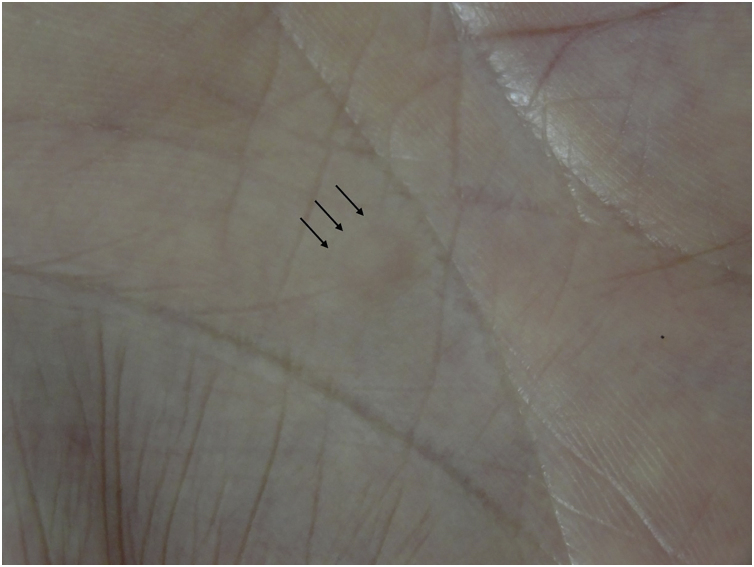
Skin-colored firm nodule on the palm (arrows).

**Figure 3:**
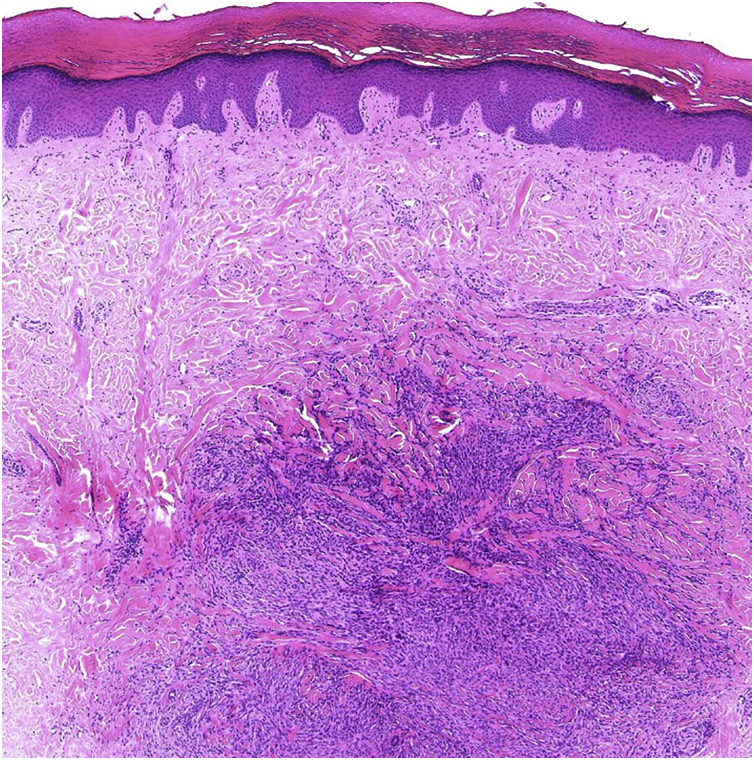
Histopathological features showing proliferation of fibroblastic cells in the dermis.

DF often develops due to fibroblastic cell proliferation as a reactive process in response to some stimuli,^2^, ^3^ and cases of digital DFs have been reported.^1^ By contrast, there have only been a few reported cases of fibrous histiocytoma occurring on the palms.^4^, ^5^ Although the patient denied precedent trauma, his hobby is wood carving, for which he frequently uses chisels with his dominant hand, namely the right hand. Therefore, it is possible that DF was triggered by repetitive mechanical stimuli. The patient had multiple porokeratotic plaques on the buttock, Achilles tendon, as well as trunk, which showed well-circumscribed brownish plaques with slightly elevated borders, but they were not either verrucous or hyperkeratotic. The porokeratosis lesions were treated with oral etretinate and topical corticosteroid for 5 years. The profibrotic effects of etretinate such as increasing collagen accumulation and reducing matrix metalloproteinase 1 have been reported.^6^ However, in the present case, DF occurred solitarily, and there have been no reports on multiple DFs in patients undergoing etretinate therapy. Therefore, the authors believe that DF occurred independently of etretinate intake. Finally, there are no reports of the coexistence of porokeratosis and dermatofibroma, and the coexistence of both disorders in the current case may be coincidental.

## Financial support

None declared.

## Author’ contributions

Toshiyuki Yamamoto: Approval of the final version of the manuscript; Critical literature review; Data collection, analysis and interpretation; Effective participation in research orientation; Intellectual participation in propaedeutic and/or therapeutic; management of studied cases; Manuscript critical review; Preparation and writing of the manuscript; Study conception and planning.

## Conflict of interest

None declared.
